# Cost-Related Nonadherence and Mortality in Patients With Chronic Disease: A Multiyear Investigation, National Health Interview Survey, 2000–2014

**DOI:** 10.5888/pcd17.200244

**Published:** 2020-12-03

**Authors:** Sarah C. Van Alsten, Jenine K. Harris

**Affiliations:** 1 Washington University in Saint Louis, George Warren Brown School of Social Work, Public Health, Saint Louis, Missouri; 2 University of North Carolina at Chapel Hill, Gillings School of Global Public Health, Chapel Hill, North Carolina

## Abstract

**Introduction:**

Prescription costs are rising, and many patients with chronic illnesses have difficulty paying for prescriptions. Missing or delaying medication because of financial concerns is common; however, the effects of cost-related nonadherence (CRN) on patient outcomes have not been described. Our objective was to determine if CRN is associated with higher all-cause and disease-specific mortality among patients living with diabetes and cardiovascular disease in a representative sample of US adults.

**Methods:**

We ascertained CRN, vital status, and cause of death for 39,571 patients with diabetes, 61,968 patients with cardiovascular disease, and 124,899 patients with hypertension in the 2000 through 2014 releases of the National Health Interview Survey. We used adjusted Cox proportional hazards models to estimate associations between CRN and all-cause mortality and CRN and disease-specific mortality.

**Results:**

On average, 15% of the sample reported CRN in the year before interview. After adjusting for confounders, CRN was associated with 15% to 22% higher all-cause mortality rates for all conditions (diabetes hazard ratio [HR] = 1.18; 95% CI, 1.1–1.3; cardiovascular disease [CVD] HR = 1.15; 95% CI, 1.1–1.2; hypertension HR = 1.22; 95% CI, 1.2–1.3). Relative to no CRN, CRN was associated with 8% to 18% higher disease-specific mortality rates (diabetes HR = 1.18; 95% CI, 1.0–1.4; CVD HR = 1.09; 95% CI, 1.0–1.2; hypertension HR = 1.08; 95% CI, 0.9–1.3).

**Conclusion:**

Relative to full adherence, CRN is associated with higher mortality rates for patients with diabetes, cardiovascular disease, and hypertension, although associations may have weakened since 2011. Policies that increase prescription affordability may decrease mortality for patients experiencing CRN.

**Summary**

**What is already known about this topic?**

Medication nonadherence is a known risk factor for diabetes and cardiovascular disease complications. Cost of medication is the most common reason for nonadherence; however, few studies have focused on nonadherence secondary to financial need.

**What is added by this report?**

We investigated how nonadherence that results from financial barriers contributes to mortality among patients with diabetes, cardiovascular disease, and hypertension. We found that people who experienced cost-related nonadherence between 2000 and 2014 experienced 15% to 22% higher all-cause mortality and 8% to 18% higher disease-specific mortality than people who did not experience cost-related nonadherence.

**What are the implications for public health practice?**

Efforts to increase medication affordability and accessibility may reduce mortality among people living with diabetes, cardiovascular disease, and hypertension.

## Introduction

The prevalence of diabetes among US adults is 15%, and the prevalence of cardiovascular disease (CVD) is 13% (1,2). Diabetes is the seventh leading cause of death in the United States and CVD is the first leading cause (3); both are associated with substantial economic burden. Together, diabetes and CVD accounted for nearly $200 billion in US health care costs in 2013; hypertension treatment accounted for another $83 billion (4). A substantial portion of these costs are from prescription expenditures, which continue to increase at a rate of 5% to 6% annually (4).

Among people with diabetes or hypertension, cost is the most common reason for medication nonadherence, with more than two-thirds of patients skipping or delaying medication (5). Such financially motivated nonadherence behaviors, including not being able to afford needed prescriptions, are collectively known as cost-related nonadherence (CRN) (6); CRN differs from other common forms of nonadherence, such as fear of medication side effects and lack of perceived need, which are determined by material insufficiency rather than psychological factors (5).

The prevalence of CRN in the US is high, particularly among patients with chronic conditions. In total, 6% to 7% of US adults reported at least 1 form of CRN in 2014 (7). In adults with type 1 or type 2 diabetes, 25% reported rationing insulin in the previous year to manage costs, 3.2% reported rationing insulin on a daily basis, and 40% reported not discussing underuse with their physician (8,9).

Despite prevalence of CRN, few studies have investigated its implications. Generally, nonadherence is associated with greater risk for hypertension, hypercholesterolemia, and elevated hemoglobin A_1c_ levels in people with diabetes (9,10), and with greater risk for dyslipidemia and extended hospitalizations in patients with hypertension or CVD (11). Although studies have documented adverse consequences of medication nonadherence, they have not specified reasons for nonadherence (ie, nonadherence because of economic factors versus psychological factors); it is unclear how CRN contributes to these outcomes. The objective of our study was to determine whether CRN is associated with higher risk of mortality in US adults with diabetes, CVD, or hypertension.

## Methods

### Source data

During 2019 until 2020, we analyzed publicly available data from the 2000 through 2014 waves of the National Health Interview Survey (NHIS) (12). The NHIS is a cross-sectional, population-representative multistage probability sample of noninstitutionalized US adults administered annually by the National Center for Health Statistics (13). All interviews were conducted using computer-assisted personal interviews by trained US Census Bureau staff and consisted of 2 parts: 1) the core questionnaire and 2) supplemental questions. The core questionnaire assesses basic demographic information, health status, behaviors, and health care utilization. Supplemental questions vary from year to year to assess current health issues and have included topics such as in-depth health care utilization and insurance information, cancer screening, and mental health. Questions relating to CRN were introduced into the survey in 2000, and 2014 is the most recent year that mortality data are available; therefore, our study included data from the 2000 to 2014 waves only.

### Study sample

We restricted the study sample to adults aged 18 years or older with diabetes, hypertension, and/or CVD for a secondary data analysis. Diabetes diagnosis was ascertained through a single item, whether participants had been told by a medical professional that they had diabetes outside of pregnancy (n = 39,571). We operationalized CVD as a diagnosis of 1 or more of the following: heart attack (n = 16,142), angina pectoris (n = 11,064), coronary heart disease (n = 21,005); or any kind of heart condition other than coronary heart disease, angina pectoris, or a heart attack (n = 35,016), or any kind of stroke (n = 13,214). In total, 61,968 respondents reported CVD. Finally, we included participants with a diagnosis of hypertension as a third subgroup (n = 133,967).

### Measures

The primary exposure of interest was CRN. Because CRN was assessed differently in the NHIS before and after 2010, we harmonized data to generate a single dichotomous variable representing whether a participant had reported CRN in the previous year. From 2000 to 2010, we operationalized CRN as a positive response to the single item asking whether participants needed, but could not afford, medication in the previous year. From 2011 to 2014, we coded CRN as any affirmative response to items asking participants if, to save money, they had skipped medication doses, taken less medicine than prescribed, or delayed taking medicine in the last year.

**Figure 1:**
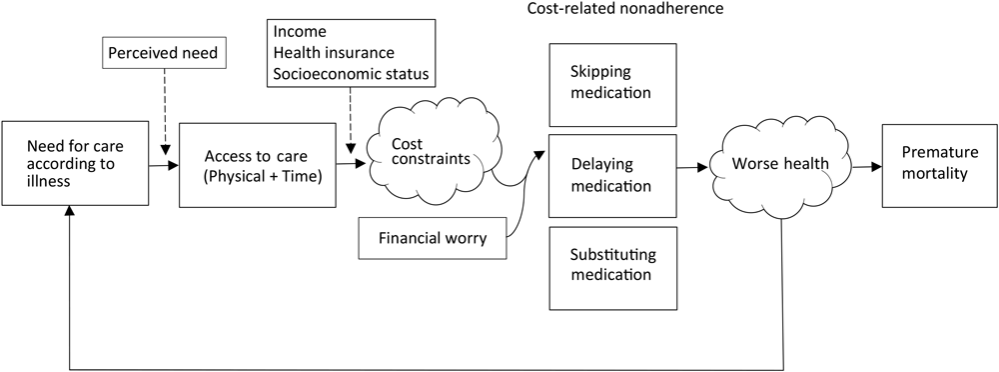
Conceptual model of the relationship between cost-related nonadherence and mortality. Boxes (measured variables) and clouds (unmeasured variables) represent determinants of health care access and utilization, eg, Andersen’s Model (15). Skipping medication means forgoing medication doses altogether as a result of cost, substitution of medication means taking cheaper alternative medications, and delaying medication means delaying taking doses or waiting to fill prescriptions to make medication last longer and save money.

The 2 primary outcomes for our analysis were all-cause and disease-specific (ie, diabetes, CVD, or hypertension) mortality. Vital status through December 2015 was determined through probabilistic linkage to the National Death Index (14). Respondents aged 18 or younger and those providing insufficient identifying information were not eligible for linkage. Follow-up time was calculated as the span between date of interview and the last day in the quarter and year of death when vital status was ascertained. For surviving participants, follow-up time was censored at December 31, 2015. We excluded 10 participants with diabetes (0.0007%) and 8 participants with heart conditions or CVD (0.0003%) from analyses because recorded death dates occurred before interview dates. No inconsistencies were noted for patients with hypertension. We operationalized all-cause mortality as any verified record of death in the National Death Index. We used a conceptual model of the factors that contribute to CRN and the proposed association between CRN and mortality (Figure 1).

#### Disease-specific mortality

The National Center for Health Statistics used probabilistic linkage between participant records and the National Death Index to determine leading and contributing causes of death for all participants with recorded mortality events. We defined disease-specific deaths due to diabetes as those for which diabetes was listed as a leading or contributing cause of death, according to the International Classification of Diseases, 10th Revision (ICD-10), codes E10–E14 (16). For CVD, we operationalized disease-specific deaths as those for which the leading or contributing cause of death was listed as heart (ICD-10 codes I00–I09, I11, I13, I20–I51) or cerebrovascular diseases (ICD-10 codes I60–I69). For hypertension, we operationalized disease-specific deaths as those for which hypertension was flagged as a contributing cause of death, as well as deaths due to essential hypertension and hypertensive renal disease (ICD-10 codes I10, I12, and I15). We defined disease-specific mortality separately by condition of interest, such that people with a history of more than 1 condition (eg, diabetes and hypertension) were considered to have the outcome in analyses only when the listed cause of death matched the primary disease of interest. For example, if patients with both diabetes and CVD had diabetes but not CVD listed as the primary cause of death, we considered them to have disease-specific morality in diabetes analyses but not in CVD analyses.

#### Statistical analyses

We compared baseline demographic characteristics of participants with and without CRN by using design-based χ^2^ tests for categorical variables and Wilcoxon signed-rank tests for continuous variables. We used Cox proportional hazard regressions to assess the associations between CRN and all-cause and disease-specific mortality among patients with diabetes, CVD, or hypertension.

**Figure 2:**
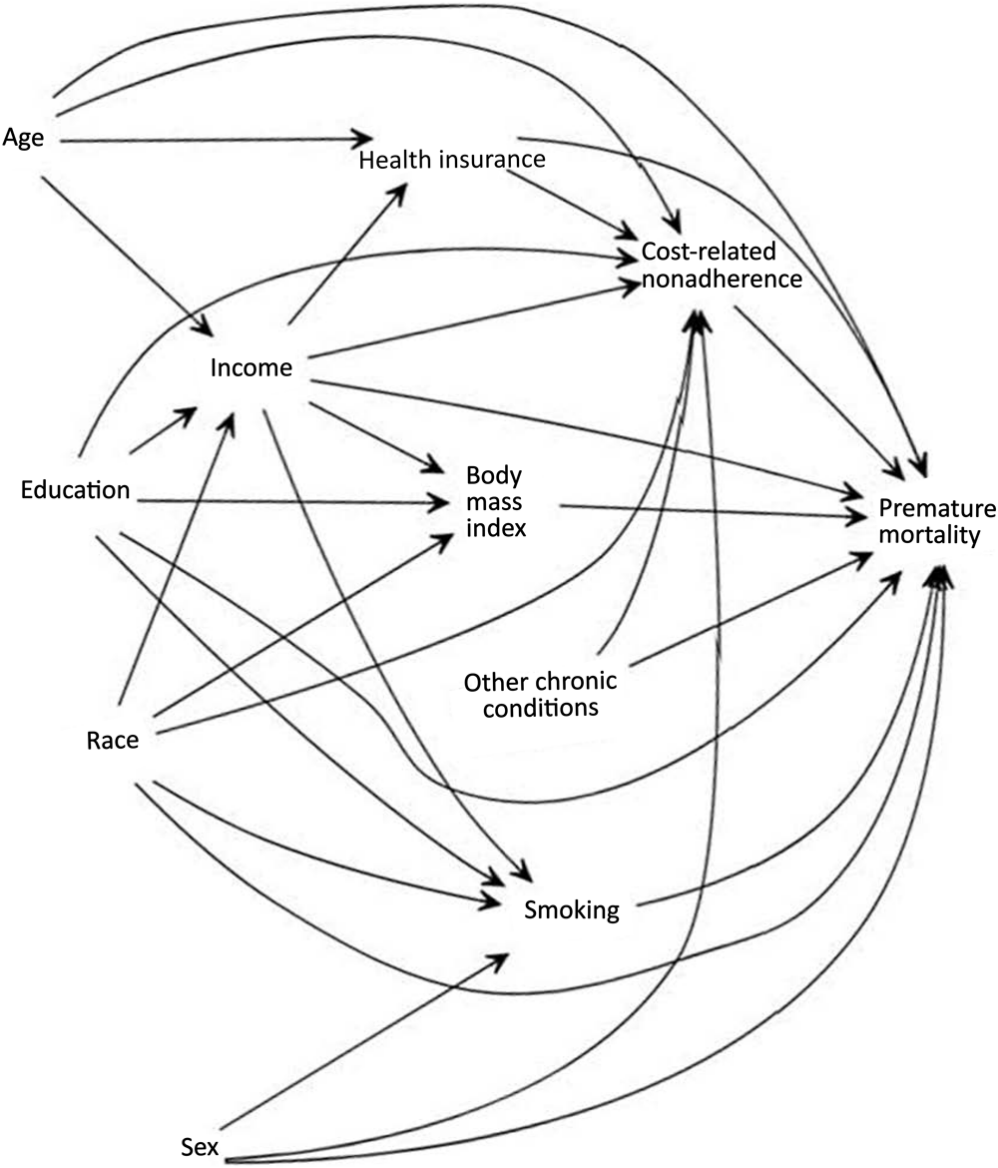
Directed acyclic graph depicting hypothesized causal interrelations between cost-related nonadherence, sociodemographic characteristics, and premature mortality.

For both all-cause and disease-specific mortality, we first fit an unadjusted model including only CRN, then adjusted for age, sex, insurance (private, public, Medicare, other, or none), race (White, Black or African American, Hispanic or Latino, Asian, or other), education (≤ high school, some college, college degree or more), and diagnoses of other chronic conditions (cancer, diabetes, hypertension, and CVD). We did not adjust for chronic conditions used to define subsamples; for example, diabetes models were adjusted for all chronic conditions except diabetes. We selected adjustment variables using a directed acyclic graph from those with a known or suspected confounding relationship between CRN and mortality (Figure 2). Unless otherwise noted, hazard estimates for CRN represent the total, rather than the direct effect of CRN on mortality.

Additionally, we conducted a sensitivity analysis by stratifying at year of interview (≤2010, >2010) to determine if the change in measurement of CRN in 2010 substantially affected findings. We evaluated models for presence of influential observations and multicollinearity by using standardized dfbeta values and variance inflation factors. We tested for proportional hazards by using scaled Schoenfeld residuals, and we assessed log-linearity by plotting Martingale residuals against continuous predictors. When models did not meet assumptions, we performed additional sensitivity analyses to assess the robustness of results against violations. For influential observations, we deleted suspected influential cases and then refit models; for log-linearity, we inspected plots for points where the log-hazard deviated from linearity, and we refit models by using natural splines at inflection points. All analyses were conducted in R, version 3.6.1 (17), and RStudio, version 1.2.5019 (18). To account for the complex sampling methodology of the NHIS, all regressions and descriptive statistics were adjusted for survey design using the survey package (19). The significance level was set at .05 throughout.

## Results

The final analytic sample sizes were 34,839 for diabetes, 53,009 for CVD, and 124,899 for hypertension. Of participants with diabetes, 15.9% reported at least 1 form of CRN, as did 15.1% of participants with CVD, and 12.7% of participants with hypertension. The most common form of CRN was needing but not being able to afford medication (88.4%–86.9% for all 3 conditions), followed by delaying medication doses (68.4%–70.3%), taking less medication than prescribed (55.7%–57.9%), and skipping medication doses (52.9%–56.2%) (Table 1). Among participants with information on specific forms of CRN, 37.8% reported all 3 CRN behaviors (delaying, taking less, and skipping medication), and 15.3% reported 2 CRN behaviors.

**Table 1:** Sociodemographic Characteristics of 2000–2014 National Health Interview Survey Participants With Diabetes, CVD, and/or Hypertension^a^

Characteristics	Reported ≥1 Form of CRN^b^			Did Not Report CRN for Combined Diabetes, CVD, and Hypertension^b^
	Diabetes	CVD	Hypertension	
Weighted no. (%)	1,872,889 (15.9)	1,895,538 (15.1)	3,749,430 (12.7)	49,886,714 (87.4)
Age, median (IQR), y	55.0 (46.0–63.0)	55.0 (45.0–65.0)	53.0 (43.0–62.0)	62.0 (49.0–73.0)
Female	61.2	62.2	62.4	54.4
**Region**				
Northeast	12.1	12.3	12.1	18.5
Midwest	23.5	24.9	22.8	24.9
South	46.1	44.7	47.6	37.8
West	18.2	18.1	17.5	18.8
**Race/ethnicity**				
White	59.9	71.5	64.5	76.1
Black	21.6	16.8	21.7	13.0
Hispanic/Latino	15.4	9.0	11.1	7.4
American Indian/Alaska Native	1.2	1.3	1.0	0.7
Asian	1.6	1.1	1.5	2.6
Other	0.3	0.2	0.3	0.2
**Health insurance coverage**				
None	25.3	25.2	30.3	6.0
Public (Medicaid, CHIP)	19.2	20.2	17.4	12.0
Private	36.7	32.7	35.5	63.6
Military	1.4	1.9	1.5	4.1
Medicare	17.1	19.7	15.0	13.9
Other	0.2	0.3	0.3	0.4
**Education**				
High school or less	58.0	55.8	56.4	49.5
Some college	30.5	32.4	32.0	27.4
≥College degree	11.5	11.8	11.6	23.2
**Household income, $**				
<20,000	45.1	50.1	46.6	26.2
20,000 to <45,000	25.4	24.2	24.6	21.1
45,000 to <65,000	19.1	16.6	18.2	23.5
65,000 to <85,000	6.2	5.3	6.2	16.8
85,000 to <100,000	1.9	1.8	2.0	4.1
≥100,000	2.3	1.9	2.3	8.4
**Cost-related nonadherence**				
Needed but could not afford medication	86.8	88.4	86.9	0
Skipped medication doses^c^	56.2	52.9	54.2	0
Delayed medication doses^c^	70.3	68.4	68.4	0
Took less medication than prescribed^c^	57.9	55.7	56.8	0

Abbreviations: CHIP, Children’s Health Insurance Program; CRN, cost-related nonadherence; CVD, cardiovascular disease; IQR, interquartile range.

a All values displayed are survey-weighted percentages unless otherwise indicated.

b All differences were significant (*P* < .001) between reporting CRN and not reporting CRN in each disease category, as determined by *t* tests or Rao–Scott χ^2^ tests.

c Indicates a survey item only included in 2010–2014 waves.

### All-cause mortality

Among participants with diabetes, 8,909 (23.6%) died of any cause during the follow-up period, 1,086 (12.2%) of whom reported CRN. The unadjusted hazard of all-cause mortality in participants with CRN was 0.75 times (HR = 0.75; 95% CI, 0.69–0.82) that of those without CRN. The direction of the association between CRN and all-cause mortality was reversed after adjustment for potential confounders, such that CRN was associated with an 18% increase in the hazard of death (HR = 1.18; 95% CI, 1.09–1.28) in people with diabetes relative to those without CRN. The unadjusted association between CRN and all-cause mortality was higher and the adjusted association was lower for participants interviewed during 2000–2010, relative to those interviewed during 2011–2014 (unadjusted *P* = .006; adjusted *P* = .004) (Table 2).

**Table 2:** Associations of All-Cause and Disease-Specific Mortality With CRN, 2000–2014 Among National Health Interview Survey Participants With Diabetes, CVD, and/or Hypertension

Disease	Follow-Up Time, Weeks, Median (IQR)	All-Cause Mortality			Disease-Specific Mortality^a^		
		Died, n (%)	Model 1, HR^b^^,^ c (95% CI)	Model 2, HR^b^^,^ d (95% CI)	Died, n (%)	Model 1, HR^b^^,^ c (95% CI)	Model 2, HR^b^^,^ d (95% CI)
**Full sample**							
Diabetes	291 (156–504)	8,909 (23.6)	0.75 (0.69–0.82)	1.18 (1.10–1.28)	3,045 (8.74)	0.77 (0.67–0.87)	1.18 (1.0–1.35)
CVD^e^	304 (160–534)	16,345 (27.8)	0.70 (0.66–0.75)	1.15 (1.07–1.23)	4,845 (9.14)	0.62 (0.55–0.70)	1.09 (0.96–1.23)
Hypertension	340 (187–539)	24,166 (19.3)	0.79 (0.75–0.84)	1.22 (1.15–1.29)	4,128 (3.30)	0.72 (0.63–0.82)	1.08 (0.94–1.25)
**2000–2010 Waves**							
Diabetes	465 (360–652)	7,379 (34.1)	0.80 (0.73–0.88)	1.22 (1.10–1.35)	2,563 (13.0)	0.81 (0.70–0.93)	1.25 (1.07–1.45)
CVD^e^	474 (330–630)	13,771 (37.4)	0.73 (0.69–0.79)	1.18 (1.10–1.27)	4,282 (12.9)	0.66 (0.58–0.75)	1.14 (1.00–1.30)
Hypertension	482 (360–635)	20,431 (27.0)	0.80 (0.76–0.85)	1.25 (1.25–1.33)	3,491 (4.60)	0.69 (0.60–0.79)	1.04 (0.89–1.21)
**2011–2014 Waves**							
Diabetes	165 (104–230)	1,530 (9.5)	0.68 (0.61–0.77)	0.97 (0.80–1.17)	482 (3.20)	0.78 (0.64–0.95)	1.02 (0.75–1.39)
CVD^e^	161 (100–230)	2,574 (11.8)	0.68 (0.61.–0.77)	0.97 (0.80–1.17)	563 (2.84)	0.78 (0.64–0.95)	1.02. (0.75–1.39)
Hypertension	174 (113–243)	3,734 (7.4)	0.67 (0.59–0.77)	1.10 (0.96–1.26)	637 (1.30)	0.68 (0.51–0.93)	1.19 (0.86–1.63)

Abbreviations: CRN, cost-related nonadherence; CVD, cardiovascular disease; HR, hazard ratio, IQR, interquartile range.

a Disease-specific mortality is defined as having a listed cause of death for diabetes, heart or cerebrovascular disease, or heart, cerebrovascular disease or underlying hypertension for diabetes, CVD, and CVD with hypertension models.

b All hazard ratios are weighted for survey design, comparing participants reporting CRN with participants not reporting CRN.

c Unadjusted hazard ratio.

d Hazard ratio adjusted for age, sex, health insurance coverage, race, education, and diagnoses of other chronic conditions: cancer (all models), diabetes (CVD and hypertension models), hypertension (diabetes and CVD models), and CVD (diabetes and hypertension models).

e The definition of CVD includes heart attack, angina pectoris, coronary heart disease, other heart condition, or stroke.

The median length of follow-up for participants with CVD was 304 weeks (IQR, 160–534). During that time, 16,345 (27.8%) of participants died CVD, 1,645 (10.1%) of whom reported CRN. CRN was associated with a 30% lower hazard of death (HR = 0.70; 95% CI, 0.66–0.75) in the unadjusted model and a 15% increase in the hazard of death after adjustment for confounders (HR = 1.15; 95% CI, 1.07–1.30) among participants with CVD. The unadjusted hazard ratio of all-cause mortality for CRN was lower among those interviewed during 2011–2014 compared with those interviewed during 2000–2010 (*P* < .001), but the adjusted hazard ratio did not significantly differ by interview period (*P* = .15) (Table 2).

During median follow-up period of 340 weeks, 24,166 (19.3) of participants with hypertension died, 2,341 (9.7%) of whom reported CRN. CRN was associated with at 21% lower hazard of death (HR = 0.79; 95% CI, 0.8– 0.8) in the unadjusted model and a 22% higher hazard of death (HR = 1.22; 95% CI, 1.2–1.3) after adjustment for potential confounders. Both the unadjusted (*P* < .001) and adjusted (*P* = .04) associations differed significantly by interview period, with higher CRN-mortality associations observed during 2000–2010 (Table 2).

### Disease-specific mortality

Among participants with diabetes, 3,045 (8.7%) died of diabetes during the follow-up period and of these participants, 392 (12.9%) reported CRN. CRN was associated with a 23% lower hazard of diabetes-related deaths before adjustment for confounders (HR = 0.77; 95% CI, 0.7–0.9). After adjustment, the direction of association changed, such that CRN was associated with an 18% higher hazard of diabetes-related death (HR = 1.18; 95% CI, 1.04–1.35). The strength of association between CRN and diabetes-related mortality did not differ between those interviewed during 2000–2010 compared with those interviewed during 2011–2014 in either unadjusted (*P* = .21) or adjusted (*P* *=* .28) models, although the association was significant only among those interviewed in the 2000 through 2010 waves.

During follow-up, 4,845 (9.1%) of participants with CVD died of heart or cerebrovascular disease, 449 (9.3%) of whom reported CRN. CRN was associated with a lower hazard of disease-specific mortality in the unadjusted model. After adjusting for confounders, participants with CVD who reported CRN had a 9% higher hazard of disease-specific mortality relative to participants who did not report CRN, although the association was not significant (HR = 1.09; 95% CI, 1.0–1.2; Table 2). When stratified by year of interview, participants who were interviewed during 2000–2010 had a significantly lower unadjusted hazard of disease-specific mortality than those interviewed during 2011–2014 (*P* *=* .02), but the adjusted hazard ratios did not differ between strata (*P =* .02).

A total of 4,128 participants (3.3%) with hypertension died with hypertension as the leading or contributing cause of death, 361 (8.7%) of whom reported CRN. In unadjusted models, CRN was associated with a lower hazard of hypertension-related deaths (Table 2). After adjusting for confounders, CRN was not significantly associated with the hazard of death (HR = 1.08; 95% CI, 0.9–1.2). We did not find evidence of heterogeneity between the 2 interview strata (unadjusted *P* = .55; adjusted *P* = .89).

### Model assumptions

Cases with suspected influence that did not substantially change estimates were deleted. Models did not show evidence of multicollinearity; all variance inflation factors were less than 1.5. Age displayed log-linearity with estimated hazards from 18–75 years, after which we found a nonlinear increase in the risks of both all-cause and disease-specific deaths. Refitting models using natural splines at age 75 years did not substantially change point estimates. Finally, although the assumption of proportional hazards was met for the CRN coefficient in all adjusted models, all models violated the proportional hazards assumption globally (*P <* .05). Schoenfeld plots did not show any clear time-dependence; therefore, these estimates were interpreted as the average hazard during the follow-up period.

## Discussion

In this secondary analysis, we found that one-eighth of persons with diabetes and CVD living in the United States reported 1 or more forms of CRN in the previous year. Although CRN was associated with lower household income and lack of health insurance, a substantial proportion of participants who were unable to afford medication had health insurance and household incomes at or above the US median. Moreover, CRN was associated with 15% to 22% higher risks of all-cause mortality among participants with diabetes, CVD, or hypertension, and 8% to 18% higher risks of disease-specific mortality among those with diabetes or CVD for all years examined, although associations were significant only for those interviewed during 2000 through 2010. Associations between CRN and mortality were of similar magnitude, regardless of illness and ultimate cause of death.

In most cases, hazards of CRN were subject to strong qualitative confounding. Considering that young people are at greater risk for CRN than older adults, and generally, at lower risk for mortality, we speculate that age played the strongest role in the reversal of association direction from unadjusted to adjusted estimates, particularly when we considered health insurance. Other confounders, such as education and higher household income, were significantly associated with mortality but were expected to be positive confounders and would likely bias estimates. For example, having a college degree or higher was inversely associated with both CRN and mortality, such that adjustment would likely move estimates closer toward the null. Sex, in contrast, was positively associated with CRN but inversely associated with mortality, such that adjustment would move estimates away from the null.

The prevalence of CRN reported in our sample (~15%) is largely consistent with assessments from other nationally representative data (20). Similarly, although research is limited on adverse outcomes associated with CRN, the association between CRN and mortality in our study is of similar magnitude to previous estimates for nonadherence generally (21), indicating that adverse effects of nonadherence might not vary according to nonadherence reasons.

In our study, the most probable mechanism for an association between CRN and mortality was through increased risk of complications. Inconsistent adherence has been shown to increase adverse outcomes in patients with diabetes and CVD, including retinopathy, nephropathy, microvascular complications (22), and uncontrolled hypertension (23). Moreover, in patients with coronary heart disease, a subtype of CVD, inconsistent adherence increases the risks of rehospitalization and receipt of a coronary revascularization procedure (23). In addition to direct effects of complications on mortality, greater disease severity often necessitates additional treatment regimens and higher financial costs, thereby potentially reinforcing the likelihood of CRN and additional adverse consequences.

Although not necessarily motivated by net cost considerations, several states have begun to consider drug pricing policies that will make medication more affordable for people with diabetes. As of April 2020, 2 states (Colorado and Illinois) had instituted a $100 monthly price limit on insulin copayments (24,25), with several others considering similar legislation. Implicit in these policies is the assumption that lower prescription drug prices will have a positive impact on patients by decreasing financial burden, improving health, or both. The common counterargument is that price controls would have negative implications for pharmaceutical research and development (26); however, these considerations should be balanced against the necessity of the drug and availability of substitutes. Continued assessment is needed to monitor policy reach, effectiveness, and potential for translation to other chronic conditions.

The major strengths of this study are the use of a large and nationally representative sample, detailed adjustment for and identification of confounding variables, and thorough robustness checks for potential threats to internal validity. The sample allowed us to investigate consequences of CRN in adults with all ranges of health insurance, including those covered through public and private sources. Previous analyses with comparable sample sizes to our study have been limited to Medicare beneficiaries only, who are not representative of the younger population and might differ on other adherence dimensions, such as positive medication values and beliefs (27,28).

This study has several limitations. First, because interviews were conducted cross-sectionally, we only had access to a single assessment of CRN, leading to probable immortal time bias in the measure of the exposure. Participants who did not report CRN at baseline may have experienced CRN later in the follow-up period and vice versa. Second, because of the change in survey questions about CRN behaviors beginning in 2011, observed differences in hazard ratios by year of interview should be interpreted with caution. Inconsistencies could be an artifact of measurement error, shorter follow-up times, differences in the distribution of confounders, or period effects such as the passage of the Affordable Care Act (29). Similarly, the measurement of CRN was nonspecific and might not have captured variations in CRN behavior with consequences for mortality. For example, respondents were also not asked about which medications that were restricted or they could not afford. Participants reporting CRN might have been adherent to crucial medications (eg, insulin, statins) and nonadherent to others (30). Lack of specificity in CRN measurement, however, would likely bias results toward the null, as we would expect that participants who were nonadherent to nonessential medications would have better outcomes than participants who were nonadherent to essential prescriptions. Third, although the number of people with improbable death dates was small and we excluded these cases from analysis, the existence of cases with erroneous (negative) lengths of follow-up time likely reduced the internal validity of mortality analyses. Nonetheless, the National Death Index is commonly used in studies of mortality and has been shown to have high sensitivity and validity when compared with other administrative records (31). Fourth, survivorship bias may exist in the CVD subsample if people who recovered from acute CVD events are overrepresented in our data. Finally, disease-specific deaths for people with diabetes or hypertension might be underestimated because of misreporting or misattribution of contributing causes of death. The degree of bias would depend on the degree to which people with CRN were misclassified relative to those without CRN.

Our results suggest that CRN is a substantial risk factor for mortality in persons with chronic illness and that efforts to address rising prescription drug costs may be valuable for improving patient health and longevity. Future studies should identify other long-term health implications of CRN and potential strategies to increase adherence in patients with limited finances to access medication.
